# Some Observations on Tuberculosis of the Nose and Pharynx
1A paper read before the Bristol Dispensary Clinical Society.


**Published:** 1912-03

**Authors:** A. J. Wright

**Affiliations:** Registrar to Special Departments, Bristol Royal Infirmary; Rhinologist, Bristol Eye Hospital


### SOME OBSERVATIONS ON TUBERCULOSIS OF THE NOSE AND PHARYNX.[1]

BY

A. J. WRIGHT, M.B., F.R.C.S.,

*Registrar to Special Departments, Bristol Royal Infirmary; Rhinologist, Bristol Eye Hospital.*

---

THE greater frequency of infection of the larynx and lungs by the tubercle bacillus has overshadowed its not very infrequent occurrence in the upper portion of the respiratory tract, the nose and pharynx. That tuberculosis of the nasal passages is not more common is no doubt due to the filter action of the vibrissæ, the ciliated epithelium, and the secretion of mucus which is distinctly bactericidal.[2] When it does occur it most commonly takes the form of lupus, the disease first showing itself on the front of the septum just inside the vestibule, probably owing to direct infection by the finger nail.

The disease follows a similar course to lupus in other parts of the body, slowly spreading and ulcerating. It is often very

[1] A paper read before the Bristol Dispensary Clinical Society.

[2] *Tr. Brit. Congr. Tuberculosis,* 1902, iii. 390.

slow in growth, but tends eventually to involve the skin of the face. It is certain that a considerable proportion of cases of facial lupus originate in the nasal passages (it has even been stated to be as many as 75 per cent. of cases), but it is only a considerable time after the nasal infection that the skin becomes involved. I have recently seen a case which well illustrates its chronic character.

The patient, an adult female, has suffered for twelve years from "nasal catarrh," with obstruction and discharge in the left side of the nose. Ten years ago she developed an acute dacryocystitis on the same side, which healed after an abscess had been opened. Now, ten years later, she has an extensive patch of lupus, involving the left side of her nasal septum and inferior turbinal, and also a few small nodules on the tip of her nose, and another patch in the scar over the lachrymal sac, infection evidently having spread up the nasal duct.

The importance of the early recognition of intra-nasal lupus is that it lends itself to free removal without leaving a visible scar, thus preventing later skin infection, and also infection of the lungs, which is not uncommon.

Cases of tuberculosis ulceration of the nasal septum also occur, differing from lupus in the more rapid and deep ulceration, with frequently the formation of masses of tuberculous granulation tissue. Ulceration of the posterior septum involving the bone is supposed to be seldom or never tuberculous, but I have recently seen two such cases. In both the condition was present in a young adult male, and in both it was apparently the only tuberculous lesion present. In each case there was a patch of ulceration over the vomer, with bare necrosed bone at its base, and with in one of them a perforation of the bony septum. The diagnosis was established by microscopical examination of portions removed. The presence of a cough, with some loss of flesh, and a positive reaction with Koch's old tuberculin, led in one of the cases to a diagnosis of phthisis with subsequent nine months' treatment in a tuberculosis dispensary and sanatorium, although at no time were there positive signs in the chest or bacilli in the sputum.

It is well recognised that tuberculosis of the pharynx occurs as a late event in phthisis as a superficial and usually very painful ulceration, involving any portion of the pharyngeal wall. In addition, however, a latent form of pharyngeal tuberculosis exists, but is not so well recognised. This condition involves the lymphoid tissue, and in a very great majority of cases that which forms the faucial tonsil. The tuberculous deposits occur deep in the tonsillar tissue round the bottom of the crypts, and remain quite latent, the tonsils as a rule undergoing shrinkage owing to fibrosis. The importance of this condition lies in the fact that a large proportion of cases of tuberculous glands in the neck are due to infection from a latent tonsillar focus. In favour of this view are the following facts :—

(1) The relative enormous frequency of involvement of the neck glands can only be explained by infection taking place through the nose, mouth, or pharynx.

(2) The gland first involved is almost invariably that one at the angle of the jaw into which the tonsillar lymphatics drain, and which is so constantly enlarged in acute tonsillar infections.

(3) Glandular infection from nasal suppurative conditions is very uncommon, and neither of the cases of tuberculosis of the nose which I have related had any enlargement of glands. The only other likely point of entry after the nose is either the lymphoid tissue of the pharynx or diseased gums and teeth. Although dental sepsis has frequently been regarded as the primary focus, it is negatived by the fact that the submental and submaxillary glands into which a large proportion of the lymphatics from the gums and teeth drain, are but seldom involved, and also that the tuberculosis of the glands in the neck does not bear any relation to the extent or existence of dental sepsis.[1]

(4) In case of slight or moderate enlargement of the glands, complete removal of the tonsils will not only check their growth, but in many cases cause their subsidence. I have observed

[1] Osborne, *Brit. M. J.*, 1911, i. 70.

twelve cases with moderate glandular enlargement varying from the size of a walnut to that of a hen's egg four to eight months after complete removal of the tonsils. In three cases the glands entirely disappeared, in seven cases the glands were just palpable, and in two they were about the same size as before; but in no case had any further growth taken place.

In another case, a boy aged nine years four years ago had a tonsil removed which was shown on section to be tuberculous. He then had considerable enlargement of the glands on the same side as the tuberculous tonsil, but the glands have now entirely subsided without further treatment.

(5) Tonsils removed from cases of tuberculosis of the neck-glands have been shown in a large proportion of cases to be tuberculous.

Thus Carmichael[1] in thirteen cases of marked enlargement of the glands found tuberculosis in the tonsil in five, although only one or two sections were cut from each tonsil; while Mathews[2] in eight cases found five of tonsillar tuberculosis, while in two more there was some other lesion in mouth or lip to account for the infection. Hurd and Wright found nine cases out of twelve examined. So that out of a total of thirty cases examined nineteen were shown to have a latent tonsillar tuberculosis.

It would seem, therefore, that in slight enlargement of the glands of the neck, in which no other primary focus can be found, and the "tonsillar" gland is the first involved, if the tonsils are completely removed the condition will not progress.

In cases of marked enlargement it would appear advisable to remove the tonsils at the same time as the glands, if an operation is necessary. It is likely that this proceeding may improve the results where operation is necessary. I find that at present out of fifty-two cases in which operation was done on the neck, nineteen have required two or more operations.

[1] *Proc. Roy. Soc. Med.*, 1910, iii. *Sect. Childr.*, p. 27.
[2] *Ann. Surg.*, 1910, lii., 753.

It would seem that removal of the primary focus should reduce this number.

The reason that the tonsil has not been recognised before as the infecting focus is that it is not only not enlarged, but actually smaller than the normal.

---

